# Risk factors associated with delayed union after open reduction and plate fixation for humeral diaphyseal fractures

**DOI:** 10.1186/s10195-025-00843-0

**Published:** 2025-05-12

**Authors:** Yuh-Ruey Kuo, Po-Yen Ko, Chun-Yi Lee, Ting-Chien Tsai, Chang-Han Chuang, Shu-Hsin Yao, Po-Ting Wu

**Affiliations:** 1 Department of Orthopedics, National Cheng Kung University Hospital, College of Medicine, National Cheng Kung University, 138 Sheng-Li Rd., North Dist., Tainan City, 704 Taiwan; 2 Department of Orthopedics, Ditmanson Medical Foundation Chia-Yi Christian Hospital, Chiayi, 60002 Taiwan; 3 Department of Orthopedic Surgery, Show-Chwan Memorial Hospital, 542, Sec 1 Chung Shan Rd., Changhua, 500 Taiwan; 4 Department of Orthopedics, College of Medicine, National Cheng Kung University, Tainan, Taiwan; 5 Department of Biomedical Engineering, National Cheng Kung University, Tainan, Taiwan; 6 Department of Biochemistry and Molecular Biology, College of Medicine, National Cheng Kung University, Tainan, Taiwan; 7 Medical Device Innovation Center, National Cheng Kung University, Tainan, Taiwan; 8 Chung Jen Junior College of Nursing, Health Science and Management, Chiayi, Taiwan; 9 Department of Orthopedics, College of Medicine, National Cheng Kung University, 1 University Road, East District, Tainan City, 701 Taiwan; 10 Department of Orthopedics, Ditmanson Medical Foundation Chia-Yi Christian Hospital, No.539, Zhongxiao Rd., East Dist., Chiayi City, 600566 Taiwan; 11 Orthopedics Department, Tainan Hospital, Ministry of Health and Welfare, Tainan, Taiwan

**Keywords:** Humeral shaft fracture, Surgical intervention, Plate osteosynthesis, Delayed union, Risk factors, Open reduction and internal fixation

## Abstract

**Background:**

The risk factors related to delayed union in humeral diaphyseal fractures (HDFs) following surgical osteosynthesis remain unclear. Therefore, this study aimed to evaluate radiological outcomes and the risk factors associated with delayed union in a retrospective cohort of patients who underwent open reduction and plate fixation (ORPF) for acute HDFs.

**Materials and methods:**

Consecutive patients with AO/OTA 12-A and AO/OTA 12-B fractures who underwent ORPF using standard compression techniques between 2017 and 2020 were enrolled in the study. Demographic data, along with serial medical records and radiographs, were collected. The included patients were divided into two groups: the timely union (union occurring within 6 months postoperatively) and the delayed union group (union occurring between 6 and 12 months postoperatively). Differences between the groups were examined, and logistic regression was subsequently applied for risk factor analysis.

**Results:**

Sixty-five cases were included in the study, consisting of 34 males and 31 females, with a median age of 38.9 years. Among these, 45 cases (69.2%) were classified in the timely union group, while 20 cases (30.8%) were classified in the delayed union group. Overall, 30 cases (46.2%) demonstrated secondary bony union. Significant differences were observed between groups in terms of fracture pattern, immediate postoperative fracture gap, union pattern, and complication rate (*p* < 0.05 for all comparisons). Multivariate logistic regression analysis revealed that the use of interfragmentary screw and the presence of postoperative complications were independent predictors of delayed union, with an adjusted odds ratio of 0.14 and 5.76, respectively.

**Conclusions:**

In ORPF for acute HSFs, 30 out of 65 cases demonstrated secondary bone union despite the use of standard compression techniques. The application of interfragmentary screws significantly reduces the risk of delayed union. Conversely, the presence of postoperative complications is associated with an increased likelihood of delayed union.

Level of evidence: 3

*Trial Registration* All procedures were approved by the institutional review board of the authors’ hospital (IRB nos. A-ER-112-395 and IRB20230089)

**Supplementary Information:**

The online version contains supplementary material available at 10.1186/s10195-025-00843-0.

## Introduction

Humeral diaphyseal fractures (HDFs) account for approximately 1–5% of all long bone fractures [1–3]. Nonoperative management is still considered the standard of care for these injuries [4, 5]; however, a higher rate of nonunion has been reported with conservative treatment (27%) compared with operative intervention (4%) [6–8]. Earlier surgical fixation may be recommended for selected patients to prevent poor outcomes and nonunion [4, 5]. Open reduction and plate fixation (ORPF) allows direct reduction and compression of the fracture gap and is regarded as a cost-effective option by many surgeons [4, 5, 9]. Minimally invasive plating, intramedullary nailing, and external fixation are other validated treatment options that have been shown to yield favorable results [10–15].

Despite successful surgical fixation, delayed union and nonunion remain significant concerns during the fracture-healing process, with reported rates as high as 33% and 8%, respectively, in the literature [6–8]. Predictors of nonunion following surgical fixation include advanced age, osteoporosis, obesity, alcoholism, smoking, the use of nonsteroid anti-inflammatory drug (NSAID), American Society of Anesthesiologists (ASA) score, and time to surgical intervention [16–19]. In addition, factors such as fracture comminution, open fracture, unstable fixation, and residual fracture gaps after fixation have also been implicated in the occurrence of nonunion [6, 20]. However, the risk factors associated with delayed union remain unclear due to a lack of related literature. Understanding these factors is beneficial for trauma surgeons in preoperative planning. Therefore, we conducted a retrospective review of all patients undergoing ORPF for acute HDFs at two hospitals. The aims of this study were to report the radiological outcomes of surgically treated HDFs and to evaluate the risk factors associated with delayed union.

## Materials and methods

### Patients

The study was approved by the institutional review board of the authors’ hospital (IRB nos. A-ER-112-395 and IRB20230089). This was a retrospective multicenter cohort study. Patients with acute diaphyseal humeral fractures (AO/OTA 12-A and AO/OTA 12-B fractures) that occurred within 3 weeks [6] who underwent open reduction and plate fixation between January 2017 and December 2020 in two tertiary referral hospitals were enrolled on the basis of the electronic surgical database in the authors’ hospitals. AO/OTA 12-C fractures were excluded from the study, because minimally invasive plating or nailing was preferred rather than ORPF. Moreover, patients with periprosthetic fractures, pathological fractures, revision surgeries, or incomplete medical records were excluded. A total of 112 cases of humeral shaft fracture were initially collected on the basis of electronic surgical database. After applying inclusion and exclusion criteria, 88 cases were enrolled. In this cohort, 10 cases were excluded due to nonunion and 13 cases were excluded due to loss of follow-up. Finally, 65 cases were included in the final analysis. All osteosynthesis procedures were performed using compression techniques including interfragmentary screw and/or compression plate techniques, with the aim of achieving primary bone union. Follow-up for all patients continued until bony union was achieved or nonunion was identified.

### Plate osteosynthesis

Under general anesthesia, patients were positioned either supine or in lateral decubitus position, depending on the selected surgical approach, which could be either anterolateral or posterior. After aseptic draping, a standard anterolateral or posterior approach was implemented according to the operating surgeon’s preference. In cases where the posterior approach was chosen, the radial nerve was carefully dissected and isolated and protected throughout the procedure [21]. Conversely, if the anterolateral approach was selected, although the radial nerve was not routinely isolated, it was ensured that neither the plate nor the screws would compromise the integrity of the nerve [22]. The fracture sites were then exposed, and the hematoma was cleaned and irrigated. Under intraoperative fluoroscopy, reduction and provisional fixation were performed using reduction clamps and, when possible, interfragmentary screws. A conventional 4.5-mm nonlocking dynamic compression plate (DCP) or a locking 4.5-mm dynamic compression plate was applied. Stability and alignment were confirmed through fluoroscopy. The wound was copiously irrigated with normal saline and then closed in layers and dressed appropriately.

### Clinical parameters

Patients’ demographic data and common comorbidities were collected, including age, gender, body mass index (BMI), presence of hypertension (HTN), diabetes mellitus (DM), alcohol and cigarette consumption, and ASA score [23]. The fracture pattern was classified according to AO/OTA classification, and the presence of open fracture was assessed using the Gustilo–Anderson classification. Surgical parameters were collected, including time between presentation and surgery, the chosen surgical approach, intraoperative blood loss, and operative duration. Implant and fixation characteristics were documented, including the total number of bicortical screws and the presence of interfragmentary screws. Postoperative complications were tracked and graded according to the Clavien–Dindo classification [24]. Grade 1 complications were defined as any deviation from the normal postoperative course without the need for pharmacological treatment or surgical, endoscopic, and radiological interventions. Grade 2 complications required pharmacological treatment with drugs other than those allowed for grade I complications. Grade 3 complications were defined as those requiring surgical, endoscopic, or radiological intervention. Grade 4 was defined as life-threatening complications. Grade 5 complications indicated patient mortality.

### Radiographic outcomes

Based on follow-up plain radiographs, union time and bone healing pattern were recorded. Union was defined as the presence of cortical bridging bone formation across at least three out of four cortices, as observed on standard orthogonal radiographs and pain-free activity [6]. Timely union is defined as union achieved within 6 months postoperatively [25], while delayed union is defined as union between 6 and 12 months postoperatively [26]. If union is not achieved within 12 months, accompanied by no further fracture healing or the need for intervention, it is classified as nonunion [8, 13, 27]. For the healing pattern, primary union was defined as the absence of callus on serial radiographs, and secondary union was defined as the presence of callus formation [28]. The quality of reduction and the remaining gap/angulation immediately after the operation were also documented [16, 29]. Reduction quality was classified as excellent if the residual gap was less than 2 mm, good if the gap was between 2 and 5 mm, and poor if the residual gap exceeded 5 mm [29]. Radiographs were reviewed by two independent orthopedic surgeons who were blinded to the clinical information. Any discrepancies in the assessment of union status were resolved through consensus with a senior surgeon.

### Statistical analysis

For evaluating the between-group differences, the normality assumption for all continuous variables were assessed using the Kolmogorov–Smirnov test. For the continuous data that are not normally distributed, values are presented as median with interquartile range (Q1, Q3) and analyzed using the Mann–Whitney *U* test. For those that are normally distributed, values are presented as mean ± standard deviation and analyzed using the Student’s *t*-test. For the comparison of the proportion, the data are presented with number (percentage) and analyzed with the chi-square test or the Fisher’s exact test when appropriate. To evaluate the risk factors associated with delayed union, the crude and covariate-adjusted odds ratios (ORs) and 95% confidence intervals (CIs) for the selected baseline variables and covariates were estimated using binary logistic regression. A *p*-value of less than 0.05 was considered statistically significant. The data were analyzed using the SPSS statistical package (version 22.0; SPSS, Chicago, IL, USA).

## Results

### Demographic data

**Fig. 1:**
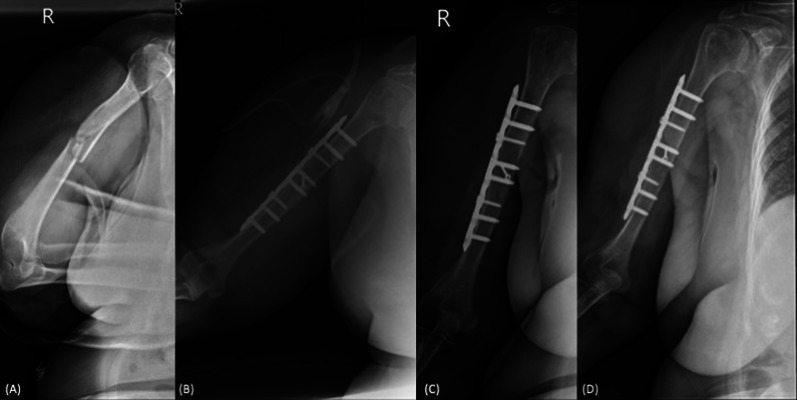
A case demonstration of the timely union group. A 73-year-old female presented with AO/OTA B2-type humeral shaft middle third shaft fracture after a scooter accident (**A**). Open reduction and plate fixation with the interfragmentary screw technique was performed. The postoperative radiography revealed the anatomical reduction (**B**). During serial follow-ups, the primary bone healing process was noted (**C**), and bony union was achieved at 3 months postoperatively (**D**)

**Fig. 2:**
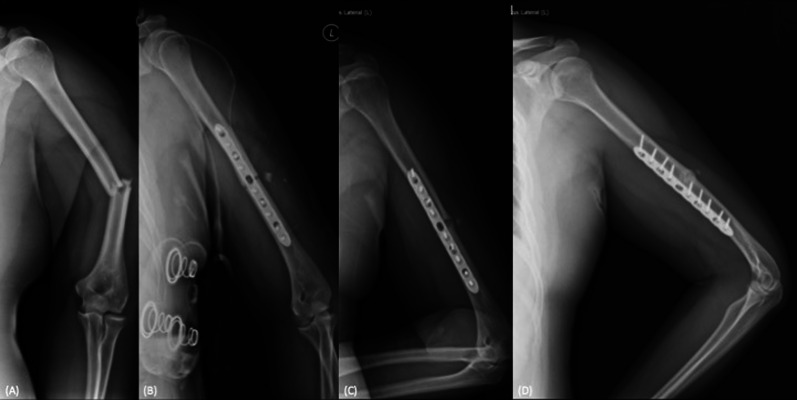
A case demonstration of the delayed union group. A 29-year-old male presented AO/OTA A3-type humeral shaft middle third fracture after a motorcycle accident (**A**). Open reduction and plate fixation with the dynamic compression technique was performed. The postoperative radiography revealed the anatomical reduction (**B**). During serial follow-ups, the secondary bone healing process was noted (**C**), and bony union was achieved at 11 months postoperatively (**D**)

Sixty-five cases, consisting of 34 males and 31 females, were included in the final analysis. The median age at the time of intervention was 38.9 years. Among the included cases, there were 5 open fractures, 46 AO/OTA A-type fractures, and 19 AO/OTA B-type fractures. Timely union occurred in 45 of the 65 cases (69.2%, Fig. 1), while delayed union was observed in 20 cases (30.8%, Fig. 2). The median union time in the delayed union group was 8.5 months (first and third quantiles: 8.0, 11.5), compared with 4.0 months (first and third quantiles: 3.0, 4.0) in the timely union group (*p* < 0.001). When comparing the two groups, significant differences were noted in the AO/OTA classification of fracture patterns, immediate postoperative fracture gaps, union patterns, and complication rates (*p* < 0.05 for all). However, there were no significant differences between the groups in terms of gender, age, underlying comorbidities, smoking, alcohol consumption, ASA score, reduction quality, immediate postoperative angulation, and other demographic variables (Tables 1 and 2). Despite direct reduction with the compression technique when the morphology of the fracture allowed, primary union was achieved in 33 of 45 cases (73.3%) in the timely group and in 2 of 18 (26.7%) in the delayed group (Table 2).

**Table 1:** Demographic data by study group

	Overall	Union		*p* value
		Delayed (*n* = 20)	Timely (*n* = 45)	
Gender				0.291
Male	34 (52.3%)	8 (40.0%)	26 (57.8%)	
Female	31 (47.7%)	12 (60.0%)	19 (42.2%)	
Age (years)	36.0 (24.0, 52.0)	37.0 (27.0, 48.5)	36.0 (24.0, 55.0)	0.881
BMI (kg/m^2^)	24.7 (21.9, 28.4)	24.5 (21.5, 29.4)	26.0 (22.5, 27.9)	0.712
Hypertension	10 (15.4%)	3 (15.0%)	7 (15.6%)	1.000
Diabetes mellitus	7 (10.8%)	1 (5.0%)	6 (13.3%)	0.423
Active smoker	8 (12.3%)	2 (10.0%)	6 (13.3%)	1.000
Alcohol consumption	4 (6.2%)	2 (10.0%)	2 (4.4%)	0.581
ASA grade				0.982
~0–2	47 (72.3%)	15 (75.0%)	32 (71.1%)	
~3–4	18 (27.7%)	5 (25.0%)	13 (28.9%)	
Time to surgery (day)	1.0 (0.3, 1.0)	1.0 (0.3, 1.5)	1.0 (0.3, 1.0)	0.726

Timely union: union within 6 months postoperatively

Delayed union: union between 6 and 12 months postoperatively

ASA, American Society of Anesthesiology

Values are presented as median with IQR (Q1, Q3) or number (percentage)

*p*-value, based on the Mann–Whitney *U* test for continuous variables and the chi-square test or the Fisher’s exact test when appropriate for the proportion

**Table 2:** Clinical parameters and radiographic outcome data by study group

	Overall		Union		*p* value
			Delayed (*n* = 20)	Timely (*n* = 45)	
Surgical approach					0.838
Posterior	19 (29.2%)		5 (25.0%)	14 (31.1%)	
Anterolateral	46 (70.8%)		15 (75.0%)	31 (68.9%)	
Blood loss (ml)		180.5 ± 163.7	144.5 ± 102.6	193.6 ± 179.8	0.559^^^
Surgical time (min)	130.0 (99.5, 195.0)		130.0 (84.0, 187.0)	127.5 (112.0, 189.0)	0.607^+^
AO/OTA classification					0.023
A1/2	24 (37%)		3 (15.0%)	21 (46.7%)	
A3	22 (33.8%)		11 (55.0%)	11 (24.4%)	
B	19 (29.3%)		6 (30.0%)	13 (28.9%)	
Open fracture (yes)	5 (7.7%)		1 (5.0%)	4 (8.9%)	1.000
Reduction quality					0.239
Excellent (< 2 mm)	47 (72.3%)		12 (60.0%)	35 (77.8%)	
Good (2–5 mm)	18 (27.7%)		8 (40.0%)	10 (22.2%)	
Poor (> 5 mm)	0		0	0	
Gap (mm)	1.4 ± 1.1		1.8 ± 1.1	1.2 ± 1.1	0.013^^^
Angulation (°)	0.0 (0.0, 0.0)		0.0 (0.0, 0.2)	0.0 (0.0, 0.0)	0.431^+^
Locking plate (yes)	61 (93.8%)		20 (100.0%)	41 (91.1%)	0.303
Both-sided 8 cortex	39 (60.0%)		12 (60.0%)	27 (60.0%)	1.000
Total screw no. on plate (≥ 8)	40 (61.5%)		12 (60.0%)	28 (62.2%)	1.000
Interfragmentary screws (yes)	21 (32.3%)		3 (15.0%)	18 (40.0%)	0.089
Complications					0.046
No	48 (73.8%)		11 (55.0%)	37 (82.2%)	
Yes	17 (26.2%)		9 (45.0%)	8 (17.8%)	
Union time (months)	4.0 (3.0, 7.5)		8.5 (8.0, 11.5)	4.0 (3.0, 4.0)	< 0.001^+^
Union pattern					< 0.001
Primary	35 (53.8%)		2 (10.0%)	33 (73.3%)	
Secondary	30 (46.2%)		18 (90.0%)	12 (26.7%)	

Timely union: union within 6 months postoperatively

Delayed union: union between 6 and 12 months postoperatively

“Both-sided 8 cortex ” means that both sides of the fracture site were purchased by screws for at least eight cortices

Values are presented as median with IQR (Q1, Q3), mean ± standard deviation, or number (percentage)

*p*-value, based on the Mann–Whitney *U* test (if not distributed normally) or Student’s *t*-test (if distributed normally) for continuous variables and the chi-square test or the Fisher’s exact test when appropriate for the proportion

^+^Variables that were not distributed normally, and shown as median (Q1, Q3)

^^^Variables that were distributed normally, and shown as mean ± standard deviation

In the overall reported postoperative complications, there were 2 cases of superficial infections that were successfully treated with oral antibiotics and 15 cases of temporary radial nerve palsy. Even though the management of the radial nerve is different in two approaches, we found that the postoperative complication rate was not significantly different between the two approaches (Supplementary Table 1).

### Risk factors associated with delayed union

For the evaluation of risk factors, all variables that exhibited significant or borderline significant differences (*p* < 0.1) between the groups were selected for logistic regression analysis to estimate the ORs and 95% CI. Fracture patterns according to AO/OTA classification were categorized into three groups: A1/A2 type, A3 type, and B type. Logistic regression analysis revealed that A3 fractures (OR 7.00, 95% CI 1.61–30.45, *p* = 0.010) compared with A1/A2, immediate postoperative fracture gap (OR 1.72, 95% CI 1.05–2.83, *p* = 0.033), and the presence of postoperative complications (OR 3.78, 95% CI 1.18–12.15, *p* = 0.025) were significant predictors of delayed union. In the multivariate logistic regression analysis, only the use of interfragmentary screws (adjusted OR 0.14, 95% CI 0.02–0.99, *p* = 0.049) and the presence of postoperative complications (adjusted OR 5.76, 95% CI 1.20–27.73, *p* = 0.029), were identified as independent factors associated with delayed union after adjusting for age, gender, and other selected variables (Table 3).

**Table 3:** Odds ratio of delayed union risk factors

Variables	Delayed union			
	Crude OR (95% CI)	*p*-value	Adjusted OR (95% CI)	*p*-value
Gender				
Male	Ref		Ref	
Female	2.05 (0.70–6.00)	0.189	2.01 (0.50–8.00)	0.325
Age (years)	1.00 (0.97–1.04)	0.903	1.01 (0.96–1.05)	0.806
AO-OTA classification				
A1 + A2	Ref		Ref	
A3	7.00 (1.61–30.45)	0.010	3.35 (0.63–17.73)	0.156
B	3.23 (0.69–15.20)	0.138	5.29 (0.72–38.60)	0.101
Gap (mm)	1.72 (1.05–2.83)	0.033	1.55 (0.88–2.70)	0.127
Interfragmentary screws (yes)	0.27 (0.07–1.04)	0.056	0.14 (0.02–0.99)	0.049
Complications				
No	Ref		Ref	
Yes	3.78 (1.18–12.15)	0.025	5.76 (1.20–27.73)	0.029

The associations between clinical characteristics and delayed union were analyzed using binary logistic regression. The results are presented as crude and adjusted odds ratios

## Discussion

In the present study, despite the utilization of standard compression techniques, the delayed union rate of acute HDFs following ORPF was still as high as 30.8%. Among the 65 cases analyzed, 30 cases (46.2%) exhibited secondary bone healing process. Significant differences were observed in AO classification, immediate postoperative fracture gap, and complication rates between the delayed and timely union groups. However, multivariate logistic regression analysis revealed that the use of interfragmentary screw was negatively associated with delayed union (adjusted OR of 0.14), while the presence of postoperative complications was positively associated with delayed union (adjusted OR of 5.76).

On average, the union time for operatively fixed HDFs has been reported to be approximately 16 weeks, regardless of whether a plate or intramedullary nail was used [13, 30]. The literature indicated that the delayed union rate ranged from 6.1% to 33.3%. Furuhata et al. noted that the delayed union rate was higher when surgical treatment occurred five or more days after injury [6]. In the present study, the delayed union rate following ORPF was found to be 30.8%, consistent with previous reports. Although several reported risk factors for nonunion, such as age, BMI, smoking and alcohol consumption, presence of open fracture, and ASA score, were assessed, they did not show statistically different differences between the delayed and timely union groups. In our cohort, ORPF using standard compression techniques aimed at achieving primary bony union was performed. However, the compression plate technique is more technically demanding compared with the interfragmentary screw technique. Notably, A3-type (transverse) fractures were the dominant fracture type in the delayed union group (Table 2), which may have a positive association with delayed union as suggested by univariate logistic analysis (Table 3).

In the multivariate logistic analysis, the impact of fracture pattern on delayed union became statistically insignificant, while the protective effect of interfragmentary screw application was confirmed. Conversely, the presence of complications, such as superficial infection and temporary radial nerve palsy, was positively associated with delayed union. This association may be related to greater soft tissue dissection involved in the surgical procedure, despite the lack of significant differences in surgical time between the groups.

ORPF is considered the preferred choice for operative osteosynthesis of acute HSFs among many surgeons [5]. Adhering to AO principles, achieving direct reduction with absolute stability and fixation is crucial in ORPF. Our results indicated that the use of interfragmentary screws plays a protective role in preventing delayed union. This phenomenon may suggest that the interfragmentary screw technique is more conductive in achieving absolute stability, compared with the compression plate technique. Despite all surgical procedures being aimed at absolute stability without the poor reduction quality, 46.2% (30/65) of cases presented secondary bone union process. Furthermore, a significant portion of patients in the delayed union group presented the secondary bone union. Therefore, the application of compression techniques, when the morphology of the fracture permits, does not guarantee primary bone union in HSFs. Furthermore, minimally invasive plate osteosynthesis (MIPO) is another option for managing closed HSFs. Some meta-analyses have reported that MIPO achieved comparable or even superior union rates compared with ORPF [9, 31–33]. Our cohort excluded cases that underwent MIPO, highlighting the need for further studies to evaluate the risk of delayed union in ORPF compared to other surgical stabilization methods.

### Limitation

This study has several limitations. First, it is a retrospective study with a limited sample size, which may affect the generalizability of the findings. Second, only ORPF aimed at achieving absolute stability were included; cases involving MIPO or intramedullary nail were excluded from our cohort. Further research is needed to evaluate the risk factors associated with delayed union in HSFs following various surgical stabilization techniques. Lastly, union was assessed solely on the basis of radiographs and chart records. Clinical outcomes, such as functional scores and range of motion, were not thoroughly evaluated.

## Conclusion

In ORPF for acute HDFs, the delayed union rate was approximately 30.8%. Thirty of 65 cases presented the secondary bone union even using the standard compression techniques. The delayed union group demonstrated a significantly higher incidence of the secondary bone union pattern. The fact that the feasible application of an interfragmentary screw significantly reduces the risk of delayed union might suggest its higher practicality than the compression plate technique in achieving absolute stability. Conversely, the presence of postoperative complications has been found to contribute to delayed union.

## Supplementary Information

**Additional file 1: Table S1.** The reported complications between two approaches. The percentage of complications between the two different approaches was analyzed using the chi-square test.

## Data Availability

The datasets used and/or analyzed during the current study are available from the corresponding author on reasonable request.

## Abbreviations

HDF: Humeral diaphyseal fracture
ORPF: Open reduction and plate fixation
NSAID: Nonsteroid anti-inflammatory drug
ASA: American Society of Anesthesiologists
DCP: Dynamic compression plate
BMI: Body mass index
HTN: Hypertension
DM: Diabetes mellitus
OR: Odds ratio
CI: Confidence interval
