# Supplementary material for: Risk factors associated with delayed union after open reduction and plate fixation for humeral diaphyseal fractures
Source: J Orthop Traumatol. 2025 May 12;26:28. doi: 10.1186/s10195-025-00843-0 (PMC12069771; doi:10.1186/s10195-025-00843-0)
Supplement: Supplementary file 1 — Additional file 1: Table S1. The reported complications between two approaches. The percentage of complications between the two different approaches was analyzed using the chi-square test. [file 10195_2025_843_MOESM1_ESM.docx]

**Supplementary Table: The reported complications between two approaches**

|  | Approaches | |  |
| --- | --- | --- | --- |
|  | Anterolateral (n=45) | Posterior  (n=19) | P value |
| Overall Complications | 11 (24.4%) | 6 (31.6%) | 0.427 |
| Radial nerve palsy | 9 (20%) | 6 (31.6%) | 0.346 |

The percentage of complications between the two different approaches were analyzed using the chi-square test.
